# Odds for Presence of Metabolic Dysfunction Associated Steatotic Liver Disease ( MASLD) in Individuals with Reported Hepatic Steatosis on Ultrasound

**DOI:** 10.12669/pjms.42.8.11294

**Published:** 2026-08

**Authors:** Pawan Kumar, Tazeen Rasheed, Faiza Sadaqat Ali, Bader Faiyaz Zuberi

**Affiliations:** 1 Pawan Kumar, FCPS, MRCP Assistant Professor, Department of Medicine/Gastroenterology, Dow Medical College, Dow University of Health Sciences, Karachi, Pakistan; 2 Tazeen Rasheed, FCPS Associate Professor, Department of Medicine/Gastroenterology, Dow Medical College, Dow University of Health Sciences, Karachi, Pakistan; 3 Faiza Sadaqat Ali, FCPS Senior Registrar, Department of Medicine/Gastroenterology, Dow Medical College, Dow University of Health Sciences, Karachi, Pakistan; 4 Bader Faiyaz Zuberi, FCPS Meritorious Professor, Consultant Gastroenterologist, OMI Hospital, Karachi, Pakistan

**Keywords:** Body mass index (BMI), Hepatic steatosis, Metabolic syndrome

## Abstract

**Objective:**

To calculate Odds Ratio for Metabolic Dysfunction Associated Steatosic Liver Disease (MASLD) in individuals with hepatic steatosis on ultrasound examination.

**Methodology:**

This case-control study was conducted in Department of Medicine, Dr. Ruth KM Pfau, Civil Hospital Karachi, Pakistan during the period 16^th^ May 2024 & 31^st^ October 2024. Patients of either gender, between the ages of 20 -70 years undergoing ultrasound were included after taking informed consent. Patients were allocated to two groups based on presence or absence of Hepatic Steatosis on ultrasound. Patients were labelled as having metabolic syndrome as defined in the operational definition. Odds Ratio was calculated for risk of metabolic syndrome in Hepatic steatosis.

**Results:**

A total of ninety-two patients/subjects fulfilling criteria were included in study, these included 37 (40.2%) males and 55 (59.8%) females. Weight and BMI were significantly higher in the Steatotic Group. Significantly higher frequencies of hypertension, metabolic syndrome and overweight were found in the Steatotic Group. Odds ratio calculation showed that the risk of metabolic syndrome in patients having hepatic steatosis was 7.273 times higher than in patients who do not have hepatic steatosis.

**Conclusion:**

Odds of developing Metabolic Dysfunction Associated Steatotic Liver Disease (MASLD) in patients who have hepatic steatosis on ultrasound are very high. Steps should be taken to prevent this complication by early identification and management.

## INTRODUCTION

In the previous few years there has been an upward trend in the prevalence of fatty liver also called Hepatic steatosis.^1^ HS is fast becoming a most significant cause of liver disease worldwide with a worldwide incidence of 47 cases per 1,000 population and is reported to be higher among males than females.^2^ It was reported that the prevalence of HS in Pakistani population is 41.37%.^3^,^4^ Fatty liver disease is segregated into alcoholic liver disease which is abbreviated as ALD and non-alcoholic fatty liver disease abbreviated as NAFLD.^5^ Term NAFLD is now replaced with Metabolic Dysfunction Associated Steatotic Liver Disease (MASLD), and it includes mild consumption of alcohol.^6^ HS is usually seen in obese and overweight individuals.

However, HS is not seen in all obese and overweight patients, and HS is also reported in people with normal weight and BMI where it is labelled as ‘non-obese NAFLD’ or ‘lean NAFLD’. In a study by Silvia Vilarinho et al showed that lean/non-obese NAFLD was diagnosed in around 20% of patients within the NAFLD population.^7^ In a Pakistani study the frequency of HS in non-obese medical students was reported at 13.7%.^8^ Criteria for grading of HS on ultrasound (US) are as under:^9^

### Grade-I:

Minimal diffuse increase in the fine echoes. Liver appears bright compared to the cortex of the kidney, with normal visualization of diaphragm and intrahepatic vessel borders.

### Grade-II:

Moderate diffuse increase in the fine echoes. Slightly impaired visualization of the intrahepatic vessels and diaphragm.

### Grade-III:

Marked increase in the fine echoes. Poor or no visualization of intrahepatic vessels and diaphragm and poor penetration of the posterior, segment of the right lobe of the liver.

HS is recognized commonly in Asians, where more precise race-related body mass index (BMI) criteria are used to determine obesity as per the WHO BMI Asian population criteria. A number of studies have been done that show that the prevalence of HS and hepatic fibrosis on liver biopsy in obese and non-obese individuals is the same.^10^ The possible associated factors with non-obese NAFLD may be visceral obesity contrary to general obesity, high fructose and high fats consumption and hereditary risk factors.^11^,^12^ Growing number of studies have revealed that Asians have a high risk of metabolic dysfunction and metabolic syndrome associated conditions including type-2 diabetes, dyslipidemia, and hypertension.^13^ In Asian population NAFLD in BMI <25 kg/m^2^ is estimated at 8-19%.^14^ In a meta-analysis by Gao Y et al, lean or non-obese NAFLD is related to a rise in the incidence of diabetes in lean individuals.^15^ It has been observed that individuals with non-obese NAFLD, possess similar cardiac and malignancy associated morbidity and death rates as compared to overweight individuals with NAFLD.

### Rationale:

Hepatic steatosis detected on ultrasonography is frequently encountered in routine clinical practice and may represent an early, readily identifiable marker of underlying metabolic dysfunction. The recent transition from NAFLD to MASLD emphasizes the central role of cardiometabolic risk factors in steatotic liver disease and highlights the need to identify affected individuals before progression to advanced liver disease or extrahepatic complications. This issue is particularly relevant in South Asian populations, where metabolic risk may occur at comparatively lower BMI thresholds and where both obese and non-obese individuals may develop hepatic steatosis. Despite the high reported burden of hepatic steatosis in Pakistan, local data quantifying the association between ultrasound-detected hepatic steatosis and MASLD/metabolic dysfunction remain limited. Therefore, this study was conducted to determine the odds of MASLD among individuals with hepatic steatosis on ultrasound, with the aim of supporting early risk stratification, timely lifestyle and metabolic interventions, and improved prevention of liver-related and cardiovascular complications.

## METHODOLOGY

This case-control study was conducted at Dr Ruth KM Pfau, Civil Hospital Karachi during the period 16^th^ May 2024 & 31^st^ October 2024. Patients satisfying inclusion/exclusion criteria were included after written informed consent. Patients of both genders between the ages of 20 years and 70 years, undergoing ultrasound abdomen were included. Patients were allocated into two groups based on presence or absence of Hepatic steatosis on US findings. Patients having HS were classified as cases and those not having hepatic steatosis were classified as controls.

### Ethical approval:

It was obtained from the Institutional Review Board of Dow University of Health Sciences. with ref. No. (IRB-3452/DUHS/Approval/2024/142 dated May 14, 2024) .

Patients consuming ≥ 30 gm/day of alcohol in males and ≥ 20 gm/day in females, those taking steatogenic medications such as methotrexate, corticosteroids etc., patients suffering from other diseases that could affect Liver Function Tests, e.g., Viral hepatitis, pregnant and nursing females, patients on parenteral nutrition, patients who have had intestinal bypass surgery for weight loss were excluded. Sample size was calculated using the reported frequency NASH of 30% in patients with fatty liver on ultrasound,^16^ sample size was calculated as 76 using power of 95% and alpha of 0.05. Sample size was calculated using PASS 2026 software.

### Operational definitions:

***Hepatic Steatosis:***^1^ On ultrasound (US): The presence of fatty liver on ultrasound (parenchymal brightness ‘the liver echogenicity will be higher than the renal cortex and spleen’.

***BMI Asian-population criteria:*** 17

**Table:** 

Underweight	<18.5
Normal	18.5–22.9
Overweight	23–24.9
Obese	≥25

### MASLD:

defined as the presence of hepatic steatosis in conjunction with at least one of the following cardiometabolic risk factor:^18^

### Data collection:

Demographic details and physical examination data were recorded of selected patients. This included age, gender, BMI, waist circumference, ALT, fasting blood sugar (FBS) and fasting lipid profile (FLP). Patients were allocated to two groups based on presence or absence of Hepatic steatosis in 1:1 ratio. Subjects without HS were called ‘Control Group’ and patients with HS were called ‘Steatosis Group’. If they were taking any anti-hypertensive, diabetic drugs or lipid lowering drugs, then test for respective disorder was not done. Patients were labelled as having metabolic syndrome as defined in operational definitions.

### Data analysis:

Frequencies of qualitative data were reported as median ±IQR and compared by χ^2^ test, quantitative data was presented as means ±SD and was compared by Student’s t-test or one-way ANOVA as per data requirements. Odds Ratio (OR) was calculated for risk of MS in HS. Sub-group analysis was done for odds ratios of components of MS and gender. For calculation of OR 2x2 table was generated using crosstab command in SPSS. OR value of >1 with 95% confidence interval on same side of ‘1’ was taken as significant. Confounders if identified, were addressed by multivariate analysis. The significance level was set at ≤.05. The analysis was done using SPSS version 27.0.1.

**Table:** 

Overweight or Obesity	BMI: ≥25 kg/m^2^ (≥23 kg/m^2^ in people of Asian ethnicity)
	Waist circumference:
	• ≥94 cm in men and ≥80 cm in women (Europeans)
	• ≥90 cm in men and ≥80 cm in women (South Asians and Chinese)
	• ≥85 cm in men and ≥90 cm in women (Japanese)
Dys-glycaemia or type 2 diabetes	Prediabetes: HbA_1c_ 39-47 mmol/mol (5.7-6.4%) or fasting plasma glucose 5.6-6.9 mmol/L (100-125 mg/dl) or 2-h plasma glucose during OGTT 7.8-11 mmol/L (140-199 mg/dl), or Type 2 diabetes: HbA_1_c ≥48 mmol/mol (≥6.5%) or fasting plasma glucose ≥7.0 mmol/L (≥126 mg/dl) or 2-h plasma glucose during OGTT ≥11.1 mmol/L (≥200 mg/dl), or Treatment for type 2 diabetes
Plasma triglycerides	≥1.7 mmol/L (≥150 mg/dl) or lipid-lowering treatment
HDL-cholesterol	≤1.0 mmol/L (≤39 mg/dl) in men and ≤1.3 mmol/L (≤50 mg/dl) in women or lipid-lowering treatment
Blood pressure	≥130/85 mmHg or treatment for hypertension

## RESULTS

A total of ninety-two patients/subjects fulfilling criteria were included in study, these included 37 (40.2%) males and 55 (59.8%) females. Comparison demographic details by Student’s t-test for age, weight, height & BMI on basis of gender revealed no statistically significant difference in age & weight but height was significantly higher in males and BMI was significantly more in females, details are in Table-I. Similar gender-based comparison of frequencies of qualitative variables by χ2 test showed significant differences in frequencies in anti-hypertensive and diabetic medicine usage, waist circumference, low HDL and impaired fasting glucose, details in Table-II. Both Steatotic Group and Control Group had 46 patients each. Weight and BMI were significantly higher in Steatotic Group (Table-III) and significantly higher frequencies of hypertension, metabolic syndrome and overweight in Steatotic Group (Table-IV).

**Table-I:** Comparison of Age, Weight, Height and BMI by gender using Student’s t-test.

	Gender			
	Male		Female	
	Mean	±SD	Mean	±SD
Age Years	32.4_a_	10.5	36.3_a_	8.4
Weight Kg	67.7_a_	14.8	69.1_a_	13.1
Height cm	166.8_a_	2.2	160.2_b_	3.5
BMI	24.36_a_	5.36	26.94_b_	5.23

***Note:*** Values in the same row and sub-table not sharing the same subscript are significantly different at p < .05 in the two-sided test of equality for column means. Tests assume equal variances.

**Table-II:** Comparison of qualitative variables by Gender by χ^2^ test.

	Gender				Sig.
	Male		Female		
	N	%	N	%	
Hepatic Steatosis	23	62.2%	23	41.8%	.056
Diabetes	25	67.6%	25	54.5%	.037*
Triglyceride Raised	21	56.8%	41	76.4%	.047*
Hypertension	23	62.2%	38	69.1%	.491
Waist Increased	27	73.0%	21	38.2%	.001*
HDL-C Low	21	56.8%	15	27.3%	.004*
IFG Present	26	70.3%	23	41.8%	.007*
Metabolic Syndrome	21	56.8%	28	50.9%	.581
Overweight	27	73.0%	41	74.5%	.866

* . The χ^2^ statistic is significant at the .05 level.

**Table-III:** Comparison of Age, Weight, Height and BMI by Hepatic Steatosis using Student’s t-test.

	Hepatic Steatosis			
	Steatotic Group		Control Group	
	Mean	±SD	Mean	±SD
Age (years)	35.6_a_	12.1	33.8_a_	5.7
Weight (kg)	73.0_a_	8.8	64.1_b_	16.2
Height (cm)	163.5_a_	4.7	162.3_a_	4.0
BMI	27.39_a_	3.66	24.42_b_	6.42

***Note:*** Values in the same row and sub-table not sharing the same subscript are significantly different at p< .05 in the two-sided test of equality for column means. Tests assume equal variances.

**Table-IV:** Comparison of qualitative variables by Hepatic Steatosis using χ^2^ test.

	Hepatic Steatosis				Sig.
	Steatotic Group		Control Group		
	N	%	N	%	
Gender	23 (M)	50.0%	14	30.4%	.056
	23 (F)	50.0%	32	69.6%	
Diabetes	28	60.9%	22	47.8%	.209
Triglyceride Raised	31	67.4%	32	69.6%	.822
Hypertension	36	78.3%	25	54.3%	.015*
Waist Increased	26	56.5%	22	47.8%	.404
HDL-C Low	17	37.0%	19	41.3%	.669
IFG Present	28	60.9%	21	45.7%	.144
Metabolic Syndrome	35	76.1%	14	30.4%	<.001*
Overweight	40	87.0%	28	60.9%	.004*

* . The χ^2^ statistic is significant at the .05 level.

The Odds Ratio was calculated for outcome of Metabolic Syndrome for exposure with Hepatic Steatosis. The result gave value of 7.273 meaning the odds of having metabolic syndrome if having hepatic steatosis is 7.273 times higher than if this exposure is not present. The details of odds ratio for other variables are given in Table-V.

**Table-V:** Odds Ratio Estimation of Metabolic Syndrome, Overweight, Hypertension, Gender, IFG, Increased Waist and Low HDL-C for Presence of Hepatic Steatosis.

Odds Ratio for Hepatic Steatosis (Steatotic Group / Control Group)	Value	95% Confidence Interval		Odds in %
		Lower	Upper	
Metabolic Syndrome	7.273	2.887	18.319	627.3%
Overweight	4.286	1.511	12.156	328.6%
Hypertension	3.024	1.218	7.510	202.4%
Gender	2.286	.974	5.367	128.6%
IFG	1.852	.808	4.243	85.2%
Waist Increased	1.418	.624	3.224	41.8%
HDL-C Low	.833	.360	1.926	16.7%

## DISCUSSION

The results of our study reveal that the risk of metabolic syndrome in patients having hepatic steatosis is 7.273 times higher than in patients who do not have hepatic steatosis. MASLD has rapidly become the most common chronic liver disease globally and is currently estimated to affect more than a third of the global adult population.^19^,^20^ It is a multisystem disease where systemic insulin resistance and related metabolic dysfunction play a pathogenic role in its development and in liver-related morbidities (cirrhosis, liver failure and hepatocellular carcinoma) and extrahepatic complications, such as cardiovascular disease (CVD), type-2 diabetes mellitus, chronic kidney disease, and certain types of extrahepatic cancers.^2^

The metabolic syndrome is a cluster of cardio-metabolic conditions, generally triggered by an expansion of the adipose visceral tissue. Previously the two-hit theory was accepted as patho-physiological explanation for MASLD. First hit was steatosis that increased the susceptibility of liver to injury mediated by cytokines, adipokines and oxidative stress. Second hit was steatohepatitis leading to fibrosis.^21^ Currently new multi-hit theory is favored which depicts multiple insults that act collectively, e.g., insulin resistance, gut microbiota, adipose tissue hormones, genetic and epigenetic factors.^22^,^23^ MASLD is the disease of current new era in human history, with sedentary life style, over eating, high caloric food intake, energy drinks, high BMI, insulin resistance and dyslipidemia more prevalent now than before. All these factors increase risk of MASLD.^24^ Much evidence is now available that MASLD is an independent risk factor of cardiovascular morbidity and mortality.^25^,^26^

In a recent systematic review and meta- analysis, it was shown that in MASLD population, pooled mortality was 12.6/1000 person-years for all-cause mortality and 4.2/1000 person-years for cardiac specific mortality.^27^ It is postulated that overproduction of glucose, VLDL, CRP, and coagulation factors by the fatty liver could contribute to the excess risk of cardiovascular disease associated with the metabolic syndrome and HS. Both of the latter conditions also increase the risk of type-2 diabetes and advanced liver disease.^28^

An observational study conducted by Goyal A et al on one hundred patients diagnosed as having metabolic syndrome according to the NCEP ATP III criteria, were subjected to ultrasonography, age and sex matched one hundred controls were also taken, and the relationship between metabolic syndrome and NAFLD was studied. The study results revealed that 73% cases of metabolic syndrome were having fatty liver, while in controls 38% persons were having fatty liver which was statistically significant.^29^ In our study weight and BMI were significantly higher in the steatotic group. It has been reported that in people with obesity, MASLD prevalence is estimated to be about 75 %.^30^ Traditionally, obesity and its related diseases have been considered a problem in Western countries. However, in the past two decades, urbanization in many Asian countries has led to a sedentary lifestyle and overnutrition, setting the stage for the epidemic of obesity.^14^

Another finding in our study was that hepatic steatosis was found to be more in males than in females. However, this was not statistically significant. The study of gender differences is a rapidly expanding area of medicine. Gender differences have been extensively studied in recent years in fields relevant to NAFLD and its pathogenesis. It has been found that in general adult populations, overall NAFLD prevalence is higher in men than in women.^31^ Sex differences also exist for the major risk factors of NAFLD. Women have a higher percentage of body fat than men. However, women tend to accumulate a lower ratio of visceral fat to subcutaneous fat. Increased visceral fat is directly associated with liver inflammation and fibrosis. Women tend to have increased subcutaneous fat which is characterized by a lower rate of lipolysis, a higher capacity for fat storage, this being associated with the release of adiponectin, protects against metabolic syndrome and MASLD.^32^

### Strengths:

It addresses a clinically relevant and emerging issue by evaluating the association between ultrasound-detected hepatic steatosis and MASLD in a local Pakistani population, using the updated MASLD terminology and Asian-specific BMI criteria. The case-control design with equal numbers of steatotic and non-steatotic participants allowed direct comparison between groups, and assessment of multiple cardiometabolic variables provided useful insight into the metabolic risk profile of patients with hepatic steatosis. The study also excluded important secondary causes of hepatic steatosis, thereby improving the clinical relevance of the findings.

### Limitations:

It was conducted at a single center with a relatively small sample size, which may limit the generalizability of the results to the wider population. The case-control design also limits the ability to establish temporality or causality between hepatic steatosis and metabolic dysfunction. Diagnosis of hepatic steatosis was based on ultrasonography, which is operator dependent and less sensitive for mild steatosis compared with advanced imaging or histological assessment. In addition, liver biopsy, elastography, insulin resistance markers, and long-term follow-up were not performed; therefore, the severity of steatohepatitis, fibrosis, and future clinical outcomes could not be assessed.

## CONCLUSION

This study has first time documented risk of MAFLD in patient with hepatic steatosis on ultrasound, by calculating odds ratio form our region. The reported risk is very high and measures should be taken to manage MASLD and to prevent its complications.

### Author’s Contribution: PK:

Literature search**,** Concept and design of study. **TR:** Critical revision of manuscript and responsible for data integrity. **FSA:** Data analysis and interpretations. Critical review. **BFZ:** Conceived study idea & gave final approval of the version to be published. All authors are responsible and accountable for the accuracy or integrity of the work.
